# Quantitative differences in antibody responses between normal donors HAM/TSP patients, asymptomatic carriers, and ATL patients from Jamaica: can they be used to estimate risk of disease?

**DOI:** 10.1186/1742-4690-8-S1-A110

**Published:** 2011-06-06

**Authors:** Yoshimi Akahata, Anna Abrams, James Goedert, Elizabeth Maloney, Steven Jacobson

**Affiliations:** 1 Neuroimmunology Branch, National Institute of Neurological Diseases and Stroke, National Institutes of Health, Bethesda, MD, USA; 2 Infections and Immunoepidemiology Branch, National Cancer Institute, Bethesda, MD, USA; 3 Division of Epidemiology, Food and Drug Administration, Silver Spring, MD, USA

## 

Adult T cell leukemia/lymphoma (ATL) and HTLV-1-associated myelopathy/tropical spastic paraparesis (HAM/TSP) are caused by HTLV-I infection. In these patients, the antibody titer and provirus load are elevated compared to levels in asymptomatic carriers (AC). These methods that determine HTLV-I infection do not differentiate between AC, HAM/TSP patients, and ATL patients. We have reported on a Luciferase Immunoprecipitation System (LIPS), a highly sensitive, quantitative technology that can efficiently detect HTLV-I antibody responses in serum of infected individuals [1]. We extended our preliminary analysis to detect anti-HTLV-I antibodies in samples from 439 persons from Jamaica: normal donors (ND), AC, ATL, and HAM/TSP patients. The antibody responses of ND differed significantly from those of HTLV-I infected patients for all three immunodominant proteins. More specifically, HAM/TSP patients were 2 times more likely to have an antibody response >1 standard deviation above the mean for ACs in gag (Odds Ratio (OR) = 2.45, 95% confidence interval [CI] = 1.23-4.88) and were 5 times more likely to exceed that threshold in env (OR = 5.27, CI = 2.51-11.08). ATL patients were 1.8 times more likely to exceed that threshold in env (OR = 1.82, CI = 1.01-3.27) and 70% less likely to exceed that threshold in tax (OR = 0.30, CI = 0.11-0.51). HAM/TSP patients had significantly higher antibody responses in gag, env, and tax compared to ATL patients. These significant differences between antibody responses in HTLV-I infected individuals may be a useful diagnostic tool in the future.
